# Suppression of Electric Field-Induced Segregation in Sky-Blue Perovskite Light-Emitting Electrochemical Cells

**DOI:** 10.3390/nano10101937

**Published:** 2020-09-29

**Authors:** Tatiana G. Liashenko, Anatoly P. Pushkarev, Arnas Naujokaitis, Vidas Pakštas, Marius Franckevičius, Anvar A. Zakhidov, Sergey V. Makarov

**Affiliations:** 1 Department of Physics and Engineering, ITMO University, 197101 St. Petersburg, Russia; zakhidov@utdallas.edu (A.A.Z.); s.makarov@metalab.ifmo.ru (S.V.M.); 2 Center for Physical Sciences and Technology, LT-10257 Vilnius, Lithuania; arnas.naujokaitis@ftmc.lt (A.N.); vidas.pakstas@ftmc.lt (V.P.); marius.franckevicius@ftmc.lt (M.F.); 3 University of Texas at Dallas, Richardson, TX 75080, USA

**Keywords:** blue perovskite LEC, solvent engineering, perovskite-polymer thin film, surface defect passivation, electric field-induced segregation

## Abstract

Inexpensive perovskite light-emitting devices fabricated by a simple wet chemical approach have recently demonstrated very prospective characteristics such as narrowband emission, low turn-on bias, high brightness, and high external quantum efficiency of electroluminescence, and have presented a good alternative to well-established technology of epitaxially grown III-V semiconducting alloys. Engineering of highly efficient perovskite light-emitting devices emitting green, red, and near-infrared light has been demonstrated in numerous reports and has faced no major fundamental limitations. On the contrary, the devices emitting blue light, in particular, based on 3D mixed-halide perovskites, suffer from electric field-induced phase separation (segregation). This crystal lattice defect-mediated phenomenon results in an undesirable color change of electroluminescence. Here we report a novel approach towards the suppression of the segregation in single-layer perovskite light-emitting electrochemical cells. Co-crystallization of direct band gap CsPb(Cl,Br)${}_{3}$ and indirect band gap Cs${}_{4}$Pb(Cl,Br)${}_{6}$ phases in the presence of poly(ethylene oxide) during a thin film deposition affords passivation of surface defect states and an increase in the density of photoexcited charge carriers in CsPb(Cl,Br)${}_{3}$ grains. Furthermore, the hexahalide phase prevents the dissociation of the emissive grains in the strong electric field during the device operation. Entirely resistant to 5.7 × 10${}^{6}$ V·m${}^{-1}$ electric field-driven segregation light-emitting electrochemical cell exhibits stable emission at wavelength 479 nm with maximum external quantum efficiency 0.7%, maximum brightness 47 cd·m${}^{-2}$, and turn-on bias of 2.5 V.

## 1. Introduction

Over the past few years, all-inorganic lead halide perovskites have gained much attention from the scientific community [1,2,3,4]. Their superior optoelectronic properties—very efficient narrow-band photoluminescence (PL) stemming from radiative recombination of room-temperature “bright” triplet excitons [5] and high charge-carrier mobilities [6]—along with the ability to tune PL over a wide spectral range [7,8] make these materials the most promising candidates for the engineering of full-color light-emitting devices [9,10]. Green and red perovskite light-emitting devices (PeLEDs) based on CsPbBr${}_{3}$ [11,12] and CsPbI${}_{3}$ [13,14,15] compositions, respectively, have recently displayed excellent electroluminescence external quantum efficiencies (EQE) as high as 20.3%. As compared to homo-halide compositions, hetero-halide (or mixed-halide) CsPb(Cl,Br)${}_{3}$ perovskites emitting blue light suffer from electric field-driven segregation [16] revealing itself in a temporal change of electroluminescence color at constant bias. However, this phenomenon always remains beyond the comprehensive description, slowing down the progress in blue PeLEDs [17,18,19,20].

The segregation in mixed-halide perovskites was first observed and further vastly studied for MAPbBr${}_{3-x}$I${}_{x}$ (MA—methylammonium) compositions. Hoke et al. [21] found structural and optical evidences of the light-evoked formation of Br-rich and I-rich perovskite domains, with the latter being acting energy funnels for charge carrier radiative recombination. Notably, structural and optical properties of the studied thin films underwent recovery in dark conditions. The major mechanism of such reversible crystal phase separation is migration of thermally activated halide ions (or their vacancies) [22,23] that could be influenced by forces arising due to the following reasons: (i) sufficient lattice strain occurring at spontaneously formed the heaviest halogen-rich (e.g., I for MAPbBr${}_{3-x}$I${}_{x}$) sites where a photogenerated polaron resides [24,25]; (ii) non-homogeneous carrier generation rate profile through the thickness of a perovskite film, that results in a directional drift of halide ions having different mobility [26]; (iii) minimizing the energy of photoexcited holes when they occupy the heaviest halogen-rich domain showing a low energy valence band edge [27]. In accordance with that, besides the general approach for mitigation of the light-evoked segregation, reducing the number of halide vacancies, there are more specific ones. Namely, one can reduce electron-phonon coupling via substituting Cs${}^{+}$ for polar MA${}^{+}$ cation, realize more homogeneous distribution of halide ions over the entire volume of perovskite, as well as more uniformly distribute excitation of this volume with a low fluence [24,25,26,27].

In contrast, the segregation dynamics in multilayer PeLEDs [16,28] is remarkably affected by strong electric field of 1 × 10${}^{6}$–10${}^{7}$ V·m${}^{-1}$ decomposing the ionic lattice of perovskite and hence producing numerous defect states, and halide vacancies in particular. This drawback should be much more pronounced in single-layer perovskite light-emitting electrochemical cells (PeLECs) with a p-i-n junction created via dissociation of perovskite into negatively and positively charged species [29,30]. From this point of view, single-layer PeLECs based on CsPb(Cl,Br)${}_{3}$ compositions should operate at extreme conditions favorable for electric field-driven segregation, whereas development of the approaches to stabilize their blue color electroluminescence would allow these materials to compete with low-dimensional monohalide counterparts [16,18,19,20,31,32,33].

In this work, we report an original solvent engineering method for the fabrication of *stabilized* blue PeLECs with a single layer derived from a mixture of poly(ethylene oxide) (PEO) and CsCl:PbBr${}_{2}$ taken in various stoichiometric ratios (1:1, 5:4, 4:3). An excess of CsCl yields polymer-assisted co-crystallization of luminescent CsPb(Cl,Br)${}_{3}$ and nonluminescent Cs${}_{4}$Pb(Cl,Br)${}_{6}$ phases. The latter passivates the surface vacancies of trihalide perovskite grains and injects extra charge carriers that result in more than 40-times increase in PL intensity of Cs${}_{4}$Pb(Cl,Br)${}_{6}$ containing films as compared to that of undoped CsPb(Cl,Br)${}_{3}$-PEO film. Furthermore, the hexahalide phase is a source of ionic species forming p- and n-type regions in the examined PeLECs and, thus, protects emissive grains from dissociation in a strong electric field. For these reasons, complete suppression of segregation is observed in ITO/CsPbCl${}_{1.22}$Br${}_{1.78}$:Cs${}_{4}$PbCl${}_{2.24}$Br${}_{3.76}$(8:1)-PEO/Ga-In structure operating at ≈5.7 × 10${}^{6}$ V·m${}^{-1}$ for 1000 s. The device exhibits sky-blue electroluminescence ($\lambda_{max}$ = 479 nm) above low turn-on bias of 2.5 V and has maximum luminance and EQE of 47 cd·m${}^{-2}$ and 0.7%, respectively.

## 2. Results and Discussion

### 2.1. Solvent Engineering

In comparison with cesium lead tribromide and triiodide, similar perovskites containing a noticeable amount of chlorine in their crystal lattice can not be tailored for depositing uniform thin films because of poor solubility of cesium(I) chloride and lead(II) chloride in organic solvents. For this reason, CsPb(Cl,Br)${}_{3}$ layer sandwiched between electrodes has numerous pinholes that lead to short-circuiting of the optoelectonic devices. A solution to this issue could come from rational solvent engineering. In particular, we reveal that injecting CsCl in methanol (MeOH, 0.158 M) and PbBr${}_{2}$ in dimethyl sulfoxide (DMSO, 0.436 M) into PEO in DMSO (20 mg·mL${}^{-1}$) results in immediate sedimentation. However, boiling this mixture at 150 ${}^{\circ }$C under mild stirring for 1 h gives a clear solution of perovskite precursors stabilized by polymer species (for details, see *Methods*). All the prepared solutions contained a fixed weight ratio of PbBr${}_{2}$ to PEO as 5:4. In the meanwhile, CsCl:PbBr${}_{2}$ ratio was varied (1:1, 5:4, 4:3) to demonstrate that an excess of the monohalide salt taken for the perovskite production reduces the concentration of halide vacancies in perovskite crystals [34,35].

### 2.2. Thin Films Morphology and Structure Description

**Figure 1:**
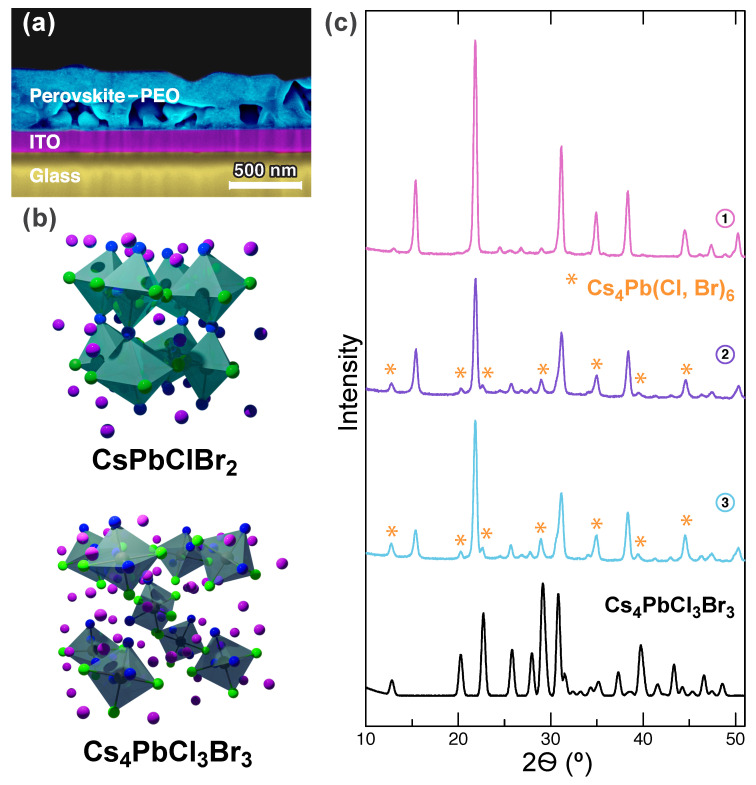
(**a**) SEM cross-sectional image of the perovskite-PEO film (**1**) on ITO substrate. (**b**) The crystal structure of an orthorhombic CsPbClBr${}_{2}$ and rhombohedral Cs${}_{4}$PbCl${}_{3}$Br${}_{3}$ phases. (**c**) XRD patterns of the samples **1**–**3** and reference Cs${}_{4}$PbCl${}_{3}$Br${}_{3}$-PEO film at room temperature. **1** shows diffraction peaks assigned to orthorhombic CsPb(Br,Cl)${}_{3}$ perovskite [8]. The patterns of **2** and **3** reveal a mixture of orthorhombic trihalide phase and rhombohedral hexahalide phase (peaks are marked by stars) that is very similar to Cs${}_{4}$PbCl${}_{3}$Br${}_{3}$.

Spin-casting of the hot composite solutions onto ITO substrates in an inert gas atmosphere, gentle evacuating the films in a vacuum chamber, and annealing them on a hotplate at ambient conditions (for details see *Methods*) provides relatively rough films with a thickness of 400–500 nm consisting of agglomerated grains and crystallites (Figure 1a and Figure S1). The films dominantly consist of tightly packed ≈200 nm size grains covered with PEO. As CsCl:PbBr${}_{2}$ ratio increases from 1:1 up to 4:3 the crystallinity of grains increases and, hence, the composite layer becomes less uniform. Furthermore, rhombohedral crystallites are clearly observed in scanning electron microscopy (SEM) images of the samples prepared with the excess of CsCl (Figure S1). These crystallites belong to indirect band gap Cs${}_{4}$Pb(Cl,Br)${}_{6}$ material. According to reaction equations (for details see Supporting Information) and wavelength of PL peak maximum (see the data below) that is dependent on halogens content in CsPb(Cl,Br)${}_{3}$ [8], the following chemical composition of inorganic components in each sample can be deduced: CsPbCl${}_{0.96}$Br${}_{2.04}$ (sample **1**), CsPbCl${}_{1.14}$Br${}_{1.86}$:Cs${}_{4}$PbCl${}_{2.46}$Br${}_{3.54}$ (11:1) (sample **2**), and CsPbCl${}_{1.22}$Br${}_{1.78}$: Cs${}_{4}$PbCl${}_{2.24}$Br${}_{3.76}$ (8:1) (sample **3**).

Coexistence of two different crystallographic phases that are stoichiometrically close to orthorhombic (Orth) CsPbClBr${}_{2}$ and rhombohedral (Rhomb) Cs${}_{4}$PbCl${}_{3}$Br${}_{3}$ (Figure 1b) is confirmed by powder X-ray diffraction (XRD). The sample **1** reveals the peaks at 2θ = 15.38, 21.84, 31.14, 34.92, 38.33 and 44.51${}^{\circ }$ (Figure 1c) that are in a good agreement with previous studies on polymer-free perovskite films [8]. In XRD patterns of **2** and **3**, these peaks descend and undergo a slight shift towards larger angles. The descending is caused by the dilution of the trihalide phase with hexahalide one. The latter is mainly presented in the patterns by new peaks at 2θ = 12.75, 20.27, 22.6, 25.7, 27.77 and 28.93${}^{\circ }$ (Figure 1c). The peaks belonging to Orth perovskite change their position because of an increase in chlorine content in CsPb(Cl,Br)${}_{3}$ structure. As consequence, an increase in bromine content in Rhomb phase leads to a slight shift of its diffraction signals towards smaller angles 2θ as compared to that of a reference Cs${}_{4}$PbCl${}_{3}$Br${}_{3}$-PEO sample (for details on thin film deposition, see *Methods*): 12.80, 20.3, 20.73, 25.82, 27.95 and 29.14${}^{\circ }$ (Figure 1c).

### 2.3. Photophysical Properties

**Figure 2:**
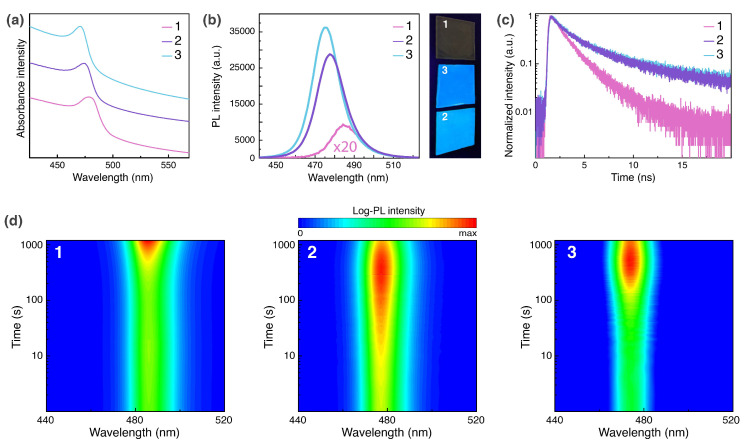
(**a**) Absorption spectra of the films **1**–**3**. (**b**) Photoluminescence spectra of the samples. The intensity of the PL peak for **1** is multiplied by 20 times. A side image: photograph of the films under evenly distributed 360 nm UV lamp illumination (*P* = 0.1 W·cm${}^{-2}$). (**c**) Time-resolved PL decay curves for **1**–**3**. (**d**) Temporal behavior of PL for the films upon severe UV excitation (*P* = 5 W·cm${}^{-2}$).

The change of a Cl:Br ratio in CsPb(Cl,Br)${}_{3}$ affects the energy of its excitonic absorption and photoluminescence. Thus, in comparison with absorption and emission peaks ($\lambda_{abs}$ = 478 nm, $\lambda_{em}$ = 485 nm, Figure 2a,b) of **1**, the spectra of chlorine-rich samples reveal a noticeable hypsochromic shift ($\lambda_{abs}$ = 474 nm and $\lambda_{em}$ = 478 nm for **2**, $\lambda_{abs}$ = 470 nm and $\lambda_{em}$ = 475 nm for **3**, Figure 2a,b). According to that, upon 360 nm excitation the perovskite-polymer films **1**–**3** emit sky-blue light with (0.107, 0.112), (0.099, 0.138), and (0.094, 0.231) CIE 1931 (x,y) chromaticity coordinates, respectively (Figure S2a). PL intensity of **2** and **3** more than 50 times surpasses the intensity of **1** that can be clearly seen when all three samples are evenly illuminated with 0.1 W·cm${}^{-2}$ UV light (side image of Figure 2b). For the comparison purpose, the image of the film **1** under 1 W·cm${}^{-2}$ excitation is shown in Figure S2b. The measured PL quantum yield (QY) values are 0.2% (**1**), 10% (**2**), and 12% (**3**). A good explanation for such a drastic difference in luminescent activity of the samples could be the passivation of the surface halide vacancies in CsPb(Cl,Br)${}_{3}$ grains by Cs${}_{4}$Pb(Cl,Br)${}_{6}$ crystallites that takes place for **2** and **3**. This hypothesis is consistent with recorded PL decay kinetics (Figure 2c). The Orth perovskite-polymer composite film shows monomolecular radiative recombination with a decay constant $\tau_{mon}$ = 1.5 ns, whereas the films containing a mixture of Orth and Rhomb phases exhibit similar temporal dynamics with a major contribution from bimolecular ($\tau_{bi}\approx$ 2.2 ns) and minor contribution from monomolecular ($\tau_{mon}$ = 12 ns) decay. Since monomolecular recombination occurs at crystal lattice defect states [36] and, at the same time, the defect states are mostly presented by surface halide vacancies (SHVs) [37] in halide perovskites the slower PL decay rate observed for **2** and **3** implies SHVs passivation in CsPb(Cl,Br)${}_{3}$ grains (Figure S3). The reduced number of SHVs in the passivated grains results in the saturation of luminescent centers at defect states sites and hence allows rapid bimolecular emission even upon low-intensity pumping ($\lambda_{ex}$ = 375 nm, *F* = 100 nJ·cm${}^{-2}$). Moreover, extra charge carriers diffuse from Cs${}_{4}$Pb(Cl,Br)${}_{6}$ into CsPb(Cl,Br)${}_{3}$ and, thus, enhance the bimolecular recombination.

Before manufacturing PeLECs and examining their EL properties, light-induced PL dynamics was studied for all the samples. Generally, the light-driven segregation in halide perovskites proceeds slowly at low optical pumping, and it is very sensitive to excitation intensity that remains a speculative factor in many reports. Therefore, the experiments were conducted in harsh conditions: the light from a continuous wave UV lamp was focused on the sample to reach *P* = 5 W·cm${}^{-2}$. As a result, the films **1**–**3** illuminated for 1000 s do not show any PL spectra shift (Figure 2d) that differs from the rapid segregation in polymer-free CsPb(Cl,Br)${}_{3}$ films [8,38]. These outcomes also confirm the passivation of SHVs since the latter assist the high-mobility ionic migration and accelerate segregation [39]. Recently, many approaches for eliminating surface defects in various mixed-halide and homohalide perovskites in order to stabilize their phases have been demonstrated among which are the passivation of the grains surface with K${}^{+}$ cations [40], Lewis base urea [41], or 4,6-di(anthracen-9-yl)-1,3-phenylene bis(dimethylcarbamate) [42], exposure of the surface to the 1-butanethiol vapor [43], oxygen treatment of a perovskite film under light illumination [44], embedding mixed-halide perovskite nanocrystals in Cs${}_{4}$Pb(Br,I)${}_{6}$ endotaxial matrix [45], and mixing 2D/3D perovskite phases [46,47]. For our samples, we assume that the partial passivation of SHVs happens due to coordinating an oxygen atom of PEO at Pb site of perovskite (Figure S3a). Apparently, one of the lone pairs of the oxygen atom neutralizes the positive charge at the Pb site. That is sufficient for preventing light-induced segregation, however additional passivation of SHVs in trihalide grains by hexahalide crystallites (Figure S3b) is required to suppress the electric field-driven segregation in PeLECs as shown below.

### 2.4. PeLECs Fabrication and Characterization

**Figure 3:**
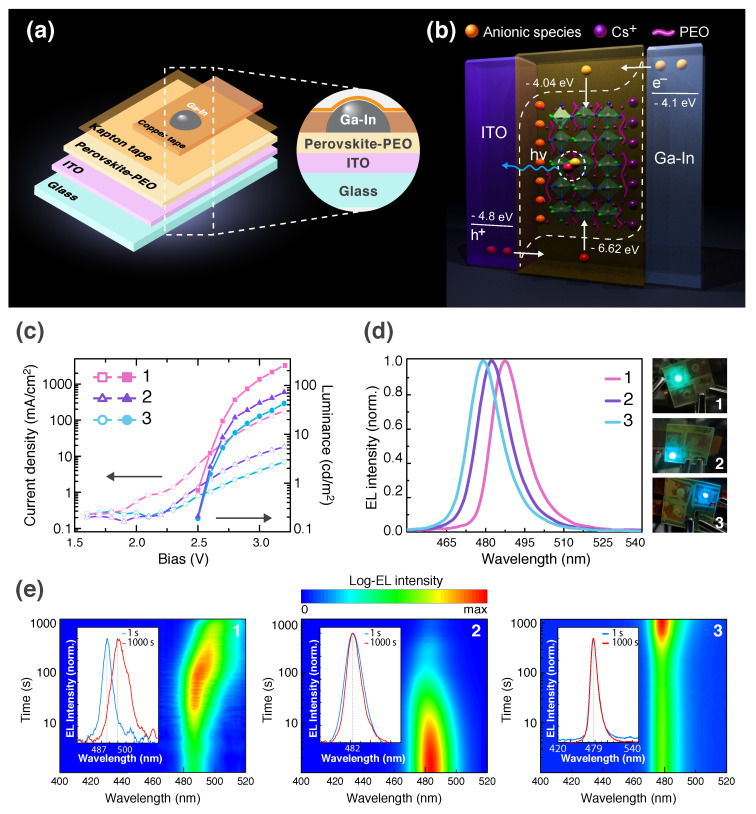
(**a**) The architecture of a PeLEC based on perovskite-polymer composite electroluminescent layer. A liquid Ga-In cathode is confined by the emissive layer, copper on insulator electrode, and Kapton tape. (**b**) The working principle of the studied PeLECs operation. Charge carriers are injected in the emissive layer from the electrodes and recombine in CsPb(Cl,Br)${}_{3}$ grains. A wide bandgap Cs${}_{4}$Pb(Cl,Br)${}_{6}$ insulating phase is omitted for clarity. (**c**) Current density–voltage and luminance–voltage relations for the devices **1**–**3**. (**d**) Normalized EL spectra and corresponding to them colors of light generated from the PeLECs operating at 3 V forward bias. (**e**) Electroluminescence temporal dynamics in the devices operating at 2.7 V bias. Inset images show EL spectra measured at the 1st (blue line) and 1000th second (red line).

For the fabrication of electroluminescent devices, a single-layer architecture, where a perovskite- PEO film is sandwiched between ITO anode and Ga-In liquid cathode, [16,48] was employed (Figure 3a). This design allows for avoiding the influence of other charge-transporting layers that are exploited in PeLEDs to increase their performance characteristics on the dynamics of the electric field-evoked segregation. To deposit a liquid electrode we inject Ga-In eutectic into a void confined by the emissive layer, copper tape, and Kapton tape (Figure 3a). Working principle of the PeLEC operation can be explained in terms of electrochemical doping model [16,30] as follows: (i) applied forward bias induces an electrostatic field in the structure that invokes dissociation of the trihalide or hexahalide phase into cationic (Cs${}^{+}$) and anionic (possibly [Pb(Br,Cl)${}_{3}$]${}^{-}$) species; (ii) these negatively and positively charged species drift in PEO towards the anode and cathode, respectively, and form p- and n-type regions in the near-electrode space; (iii) charge carriers are injected in the perovskite-polymer layer (an intrinsic region) in quasi-ohmic manner and recombine on the CsPb(Cl,Br)${}_{3}$ grains (Figure 3b).

The devices **1**–**3** have a low turn-on bias of 2.5 V that is significantly lower as compared to that of blue PeLEDs demonstrated so far. [20,32,49,50,51]. When the fraction of insulating hexahalide phase increases in the emissive layer, both current density through the p-i-n junction and electroluminescence intensity go down (Figure 3c). In the meanwhile, maximum EL EQE increases with an increase in Cs${}_{4}$Pb(Cl,Br)${}_{6}$ content: 0.17, 0.5, and 0.7% for **1**–**3**, respectively (Figure S4). It should be pointed out, that we add a small amount of Cs${}_{4}$Pb(Br,Cl)${}_{6}$ phase in our devices because of its insulating behavior. Complete passivation of trihalide grains by hexahalide crystallites prevents the charge carrier injection into the former ones and, thus, reduces their electroluminescence. Current-voltage and luminance-voltage characteristics were recorded for 10 s. During this time interval, the examined PeLECs exhibit single peak emission at 487 nm (**1**), 482 nm (**2**), and 479 nm (**3**) with a linewidth of ≈15 nm (Figure 3d) and no EL color change is observed for each device (side images of Figure 3d show colors of the generated light). The maximum brightness of the samples is reached at 3.2 V bias: 263 cd·m${}^{-2}$ (**1**), 71 cd·m${}^{-2}$ (**2**), and 47 cd·m${}^{-2}$ (**3**). Note, by comparison with PL spectra of **1**–**3**, corresponding EL spectra are slightly redshifted (by 2–4 nm). The reason for the redshift could stem from both the photons recycling in a thick emissive layer and the radiative recombination at shallow traps that are SHVs. A contribution of the trap-assisted recombination to overall electroluminescence can be roughly evaluated by fitting a Shockley–Read–Hall region (2.3–2.8 V) of the current density-voltage (*J*-*V*) curve as follows:

(1) $$J(V)=J_{0}\exp\left(\frac{qV}{\xi kT}-1\right)$$

where *J${}_{0}$* is the saturation current density, ξ is the ideality factor, *k* is the Boltzmann constant, *T* is the absolute temperature, and *q* is the electron charge. For instance, it is found out that ξ≈ 6 for the device **1** based on CsPb(Cl,Br)${}_{3}$-PEO composite. Therefore, trap-assisted recombination substantially prevails over radiative band-to-band recombination. Such a behavior is not only defined by initially existed SHVs but also by defect states generated during the p-i-n junction formation. Fitting *J*-*V* curves of **2** and **3** gives larger ideality factors of about 8 and 10, respectively. We believe that the reason for the increase in ξ is not a higher concentration of the electrically generated SHVs in **2** and **3** as compared to that of **1**. On the contrary, this is most likely due to the mixing of the conductive trihalide phase with the insulating hexahalide one increases series resistance of the composite films.

There are two general approaches that were successfully employed for the suppression of electric field-induced segregation in multilayer red PeLEDs containing 3D mixed-halide Br-I phases. The first one is utilizing ABX${}_{3}$ compositions with mixed cations at A site (e.g., FA${}_{0.83}$Cs${}_{0.17}$Pb(I${}_{0.66}$Br${}_{0.34}$)${}_{3}$, FA—formamidinium) [52]. The mixing cations can improve crystallinity of perovskite grains and hence give a thin polycrystalline film with reduced overall area of grain boundaries that always have SHVs. The second one is passivation of the defective grain boundaries with some organic halide salt (e.g., BAI${}_{1-x}$Br${}_{x}$—*n*-butylammonium iodide-bromide) [53]. For blue electroluminescent devices based on 3D Cl-Br perovskites, there is no similar study to the best of our knowledge. The PeLECs **1**–**3** were examined at 2.7 V forward bias, that corresponds to ≈5.7 × 10${}^{6}$ V·m${}^{-1}$ electric field, for 1000 s (Figure 3e). The device **1** shows EL spectrum shift from 487 to 500 nm. It is worth mentioning, that after turning off applied voltage the EL spectrum of this sample undergoes recovery in 30 min. The device **2** reveals no bathochromic shift, however, its electroluminescence intensity notably deteriorates in time. Oppositely, the sample **3** exhibits a temporary stable EL peak without any deterioration of intensity (Figure 3e). As a result, CsPbCl${}_{1.22}$Br${}_{1.78}$:Cs${}_{4}$PbCl${}_{2.24}$Br${}_{3.76}$(8:1)-PEO thin film demonstrates 3 times better resistance against electric field-driven segregation than recently reported quasi-2D (iBA${}_{x}$PEA${}_{1-x}$)${}_{2}$Cs${}_{n-1}$Pb${}_{n}$(Br${}_{0.7}$Cl${}_{0.3}$)${}_{3n+1}$ (iBA—i-butylammonium, PEA—phenylethylammonium) perovskites [54]. Since in our PeLECs segregation is not only mediated by initially existed SHVs in perovskite grains but also the crystal lattice defects generated during p-i-n junction formation accelerate anionic migration, it is not surprising that device **1** consisting of PEO and the dissociating in electric field perovskite exhibits EL spectrum change in time. Bearing this in mind, a possible mechanism for the suppression of segregation in the devices **2** and **3** is decomposing the nonluminescent hexahalide phase instead of luminescent trihalide one. This hypothesis is advocated by the fact that the melting point of ternary alkali lead hexahalides and hence their lattice energy is lower than that of ternary alkali lead trihalides [55,56,57]. The lower the lattice energy of an ionic compound, the more it tends to rapid decomposition in the strong electric field. Thus, the mixing of CsPb(Cl,Br)${}_{3}$ with Cs${}_{4}$Pb(Cl,Br)${}_{6}$ and PEO can be considered as a superior strategy for the development of segregation free perovskite electroluminescent devices.

Besides the segregation dynamics, temporal behavior of EL intensity in the studied devices needs to be explained. We assume that different dynamics of EL intensity in PeLECs **1**–**3** operated at constant bias (Figure 3e) depend on the rate of forming a p-i-n junction and expansion of its n- and p-type regions, as well as on conductivity of the emissive region. Since Cs${}_{4}$Pb(Cl,Br)${}_{6}$ phase is supposed to decompose in a strong electric field more rapidly than CsPb(Cl,Br)${}_{3}$ one does, n- and p-type regions form and expand in **2** faster than in **1**. This leads to rapid and slow increase in charge carrier injection affecting electroluminescence in **2** and **1**, respectively. As for device **3**, rapid formation of p-i-n junction does not result in a similar rapid increase in EL intensity because the intrinsic (i-type) region still contains some amount of insulating hexahalide phase providing this region with low conductivity and limiting radiative charge carrier recombination. Once the hexahalide phase is decomposed in the electric field, the emissive region becomes more conductive and generates more photons. In the meanwhile, deterioration of electroluminescence intensity in all the devices could be related to thermal destruction of luminescent perovskite material caused by high current flow.

## 3. Experimental Section

### 3.1. Materials

Lead(II) bromide (PbBr${}_{2}$, 99.998%, Alfa Aesar, Haverhill, MA, USA), cesium(I) chloride (CsCl, 99.9%, Sigma-Aldrich, St. Louis, MI, USA), cesium(I) bromide (CsBr, 99.9%, Sigma-Aldrich), dimethyl sulfoxide (DMSO, anhydrous, 99.8%, Alfa Aesar Haverhill, MA, USA), methanol (MeOH, anhydrous for analysis, max. 0.003% H${}_{2}$O, Merck, Darmstadt, Germany), poly(ethylene oxide) (PEO, M${}_{V}$ = 5,000,000, Sigma-Aldrich St. Louis, MI, USA) were used as received. ITO substrates and Ga-In eutectic were purchased from commercial suppliers.

### 3.2. Preparation of Composite Solutions

PEO (20 mg) is added to DMSO (1 mL) and the mixture is stirred (200 rpm) on a hotplate at 50 ${}^{\circ }$C overnight to afford a solution **A**. PbBr${}_{2}$ (320 mg) is dissolved in DMSO (2 mL) to give a solution **B**. CsCl (80 mg) is dissolved in MeOH (3 mL) to obtain a solution **C**. **B** (203 mg) is added to **A** (1120 mg) and the mixture (**D**) is stirred at 100 ${}^{\circ }$C for 10 min. To afford solutions for the deposition of the samples **1**–**3** a different amount of **C** is added dropwise to **D** cooled down to room temperature: 360 mg (solution **1**), 450 mg (solution **2**), and 480 mg (solution **3**). Thereafter, the cloudy solutions **1**–**3** are boiled at 150 ${}^{\circ }$C for 1 h until they become transparent. A solution for the deposition of Cs${}_{4}$PbCl${}_{3}$Br${}_{3}$–PEO film is prepared in a similar fashion: (i) CsCl (60 mg) and CsBr (25.3 mg) are dissolved in MeOH (3 mL) to give a solution **E**; (ii) 1640 mg of **E** is added dropwise to **D**; (iii) 1100 mg of DMSO is added to the resulting mixture and then it is boiled at 150 ${}^{\circ }$C for 1 h. All the chemicals are stored and mixed inside a N${}_{2}$-filled glove box with both O${}_{2}$ and H${}_{2}$O level not exceeding 1 ppm.

### 3.3. Thin Films Deposition

ITO substrates are cleaned by subsequent ultrasonication in acetone and 2-propanol. Then the substrates are rinsed with deionized water and exposed to O${}_{3}$ for 10 min to improve wettability of the surface. Hot solutions (100 ${}^{\circ }$C) are spin-casted onto the substrates at 2500 rpm for 10 min in the glove box. Then the deposited thin films are gently evacuated (−0.1 bar/min) in a vacuum chamber for 10 min. After that, the samples are annealed on a hotplate at 200 ${}^{\circ }$C for 35 s at ambient conditions.

### 3.4. Characterization of Thin Films

Surface morphology of the samples is studied using a scanning electron microscope (FE-SEM-FIB HELIOS Nanolab 650, FEI). XRD patterns of the samples are measured using an X-ray diffractometer (SmartLab, Rigaku, Tokyo, Japan) equipped with a 9 kW rotating Cu anode X-ray tube. The measurements are performed using the grazing incidence (GIXRD) method in the 2θ range of 10−50${}^{\circ }$. The angle between the X-ray primary beam and the specimen surface is adjusted to 0.5${}^{\circ }$. Optical absorption spectra are measured for similar composite films on glass substrates by using UV-vis-NIR spectrophotometer (Shimadzu, Kyoto, Japan, UV-2600). Fluorescent images of the samples are obtained on a microscope (Axio Imager.A2m, Carl Zeiss, Jena, Germany). PL spectra excited at 360 nm (HBO 100 UV lamp, Osram, Berlin, Germany) are recorded on a fibre optic spectrometer (QE Pro, Ocean Optics, Bryan Dairy Rd Largo, FL, USA) coupled with the microscope. PL QY for the films is measured by using an integrating sphere (Labsphere, North Sutton, NH, USA). Fluorescence decay kinetics are measured using the Edinburgh Instruments time-correlated single photon counting fluorescence spectrometer F900. The picosecond pulsed diode laser EPL-375 emitting 50 ps pulses at 375 nm with the repetition rate of 5 MHz was used for the sample excitation. The time resolution of the setup was about hundred of picoseconds by applying apparatus function deconvolution.

### 3.5. Device Fabrication and Characterisation

For the manufacturing of PeLECs, a piece of copper tape with a hole (0.2 cm${}^{2}$) is attached to the emissive layer and then covered with a piece of Kapton tape. Ga-In liquid electrode is injected from a syringe into the volume confined by the emissive layer, copper on insulator, and Kapton tape. The current density–voltage characteristics are measured on a sourcemeter (series 2400, Keithley, Cleveland, OH, USA) connected to PC. The luminance-voltage characteristics are measured by using a luminance meter (PKM 02, TKA, West Palm Beach, FL, USA) synchronized with the picoammeter via PC. EL EQE for the devices is measured by using the integrating sphere. EL spectra dynamics is recorded on the fibre optic spectrometer. The electrode deposition and all the measurements are conducted in the glove box.

## 4. Conclusions

In summary, we have developed sky-blue-emitting lead halide perovskite-polymer composites that are robust to both photo-induced (UV light excitation of 5 W·cm${}^{-2}$) and electric field-induced (the field strength of 5.7 × 10${}^{6}$ V·m${}^{-1}$) segregation at least for 1000 s. Introducing a small amount of nonluminescent Cs${}_{4}$Pb(Cl,Br)${}_{6}$ into CsPb(Cl,Br)${}_{3}$-poly(ethylene oxide) (PEO) composite via an in situ polymer-assisted co-crystallization of these two solids during the deposition of thin films results in the passivation of surface halide vacancies (SHVs) in CsPb(Cl,Br)${}_{3}$ grains by PEO and Cs${}_{4}$Pb(Cl,Br)${}_{6}$ crystallites. This passivation yields highly photoluminescent (PL) and free of light-induced segregation films. In the meanwhile, the hexahalide phase decomposes in the strong electric field instead of the trihalide one and produces a p-i-n junction in single-layer perovskite-polymer light-emitting electrochemical cells (PeLECs). As a result, fewer defect states mediating the segregation in electroluminescent (EL) perovskite grains are generated that is a primary reason for the temporary stable EL behavior of the engineered devices. The most efficient PeLEC with a structure ITO/CsPbCl${}_{1.22}$Br${}_{1.78}$:Cs${}_{4}$PbCl${}_{2.24}$Br${}_{3.76}$(8:1)–PEO/Ga-In exhibits stable emission at wavelength 479 nm with maximum external quantum efficiency 0.7%, maximum brightness 47 cd·m${}^{-2}$, and turn-on bias of 2.5 V. Its electroluminescence color stability is improved by 3 times [54]. We believe that our findings will expedite further progress in blue PeLECs and PeLEDs for the development of full color displays, and will promote mixed-halide perovskite-polymer composites for engineering solar-to-steam generators [58], solar cells [59], and piezoelectric nanogenerators [60].

## Supplementary Materials

The following are available online at https://www.mdpi.com/2079-4991/10/10/1937/s1. Figure S1. (**a**) Fluorescent microimages of the films 1–3. (**b**) Large-scale SEM images of the samples. (**c**) High-resolution SEM images of the samples. (**d**) Grains size distribution derived from the high-resolution SEM images. Figure S2. (**a**) CIE 1931 coordinates displaying a color of photoluminescence from 1–3. (**b**) A photograph of the sample 1 under intense UV light of 1 W·cm^−2^. Figure S3. (**a**) Partial passivation of surface halide vacancies in a perovskite grain by oxygen atoms of PEO. (**b**) More complete passivation of surface halide vacancies in a perovskite grain by both PEO and hexahalide crystallites. Figure S4. Electroluminescence external quantum efficiency vs bias relations for the PeLECs 1–3.

## Abbreviations

The following abbreviations are used in this manuscript:

EL	Electroluminescence
EQE	External quantum efficiency
PeLECs	Perovskite light-emitting electrochemical cells
PeLEDs	Perovskite light-emitting devices
PEA	Phenylethylammonium
PEO	Poly(ethylene oxide)
PL	Photoluminescence
SHV	Surface halide vacancies
