# Supplementary material for: Suppression of Electric Field-Induced Segregation in Sky-Blue Perovskite Light-Emitting Electrochemical Cells
Source: Nanomaterials (Basel). 2020 Sep 29;10(10):1937. doi: 10.3390/nano10101937 (PMC7600448; doi:10.3390/nano10101937)
Supplement: Supplementary file 1 [file nanomaterials-10-01937-s001.pdf]

Article

Suppression of electric field-induced segregation in sky-blue perovskite light-emitting electrochemical cells

Tatiana G. Liashenko [1,*,‡], Anatoly P. Pushkarev [1,*,‡], Arnas Naujokaitis [2], Vidas Pakštas [2], Marius Franckevičius [2], Anvar A. Zakhidov [1,3], Sergey V. Makarov[1]

1 Department of Physics and Engineering, ITMO University, 197101 St. Petersburg, Russia;
2 Center for Physical Sciences and Technology, LT-10257 Vilnius, Lithuania;
3 University of Texas at Dallas, Richardson TX 75080, USA;
* Correspondence: tatiana.liashenko@metalab.ifmo.ru (T.G.L.); anatoly.pushkarev@metalab.ifmo.ru (A.P.P.)
‡ These authors contributed equally to this work.

Version September 28, 2020 submitted to Journal Not Specified

---

Chemical equations:

1) $CsCl + PbBr_2 \longrightarrow CsPbClBr_2$

2) $\frac{5}{4}CsCl + PbBr_2 \longrightarrow \frac{1}{12}Cs_4PbCl_{2.46}Br_{3.54} + \frac{11}{12}CsPbCl_{1.14}Br_{1.86}$

3) $\frac{4}{3}CsCl + PbBr_2 \longrightarrow \frac{1}{9}Cs_4PbCl_{2.24}Br_{3.76} + \frac{8}{9}CsPbCl_{1.22}Br_{1.78}$

© 2020 by the authors. Submitted to *Journal Not Specified* for possible open access publication under the terms and conditions of the Creative Commons Attribution (CC BY) license (http://creativecommons.org/licenses/by/4.0/).

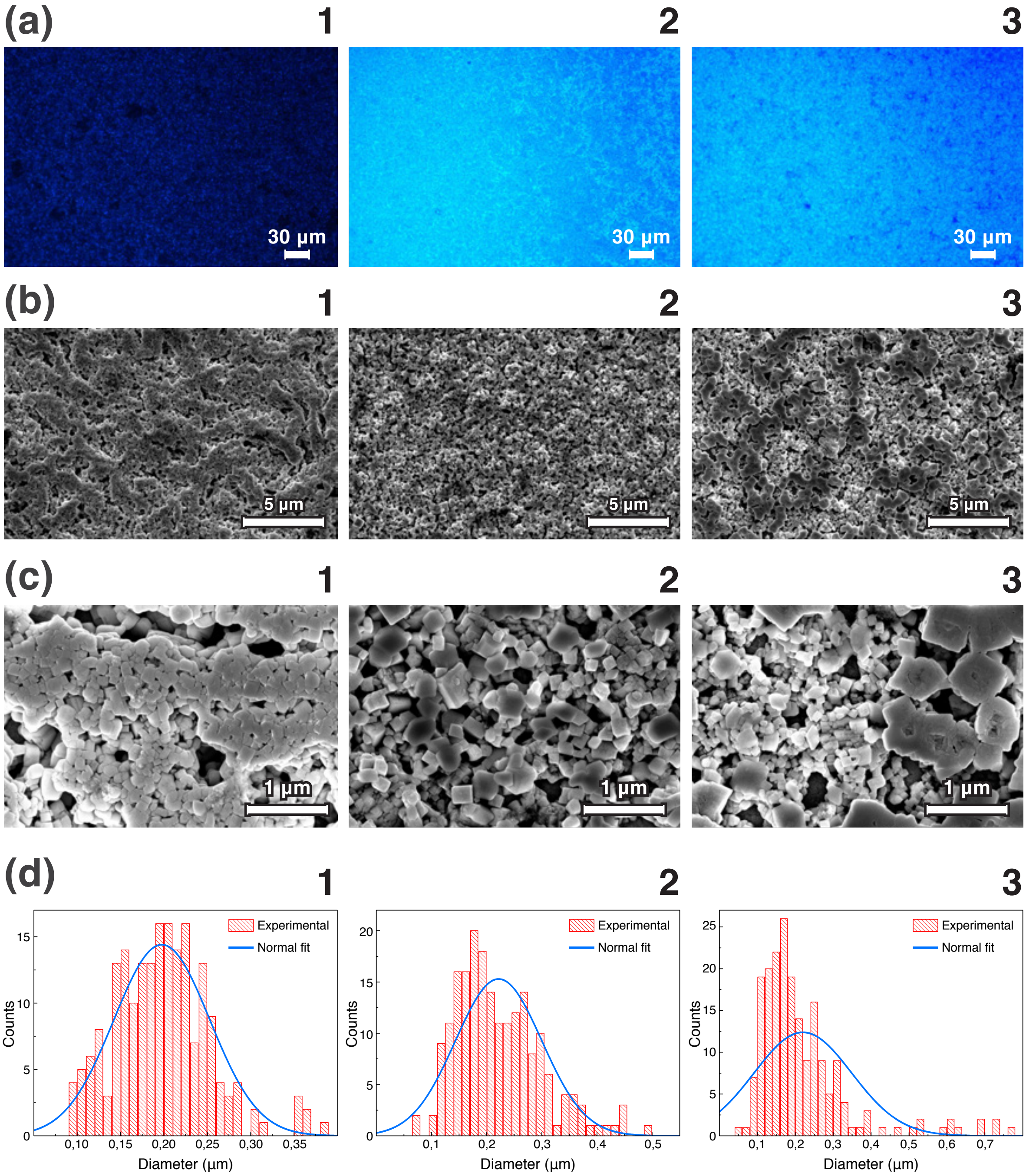

Figure S 1. (a) Fluorescent microimages of the films **1**-**3**. (b) Large-scale SEM images of the samples. (c) High-resolution SEM images of the samples. (d) Grains size distribution derived from the high-resolution SEM images.

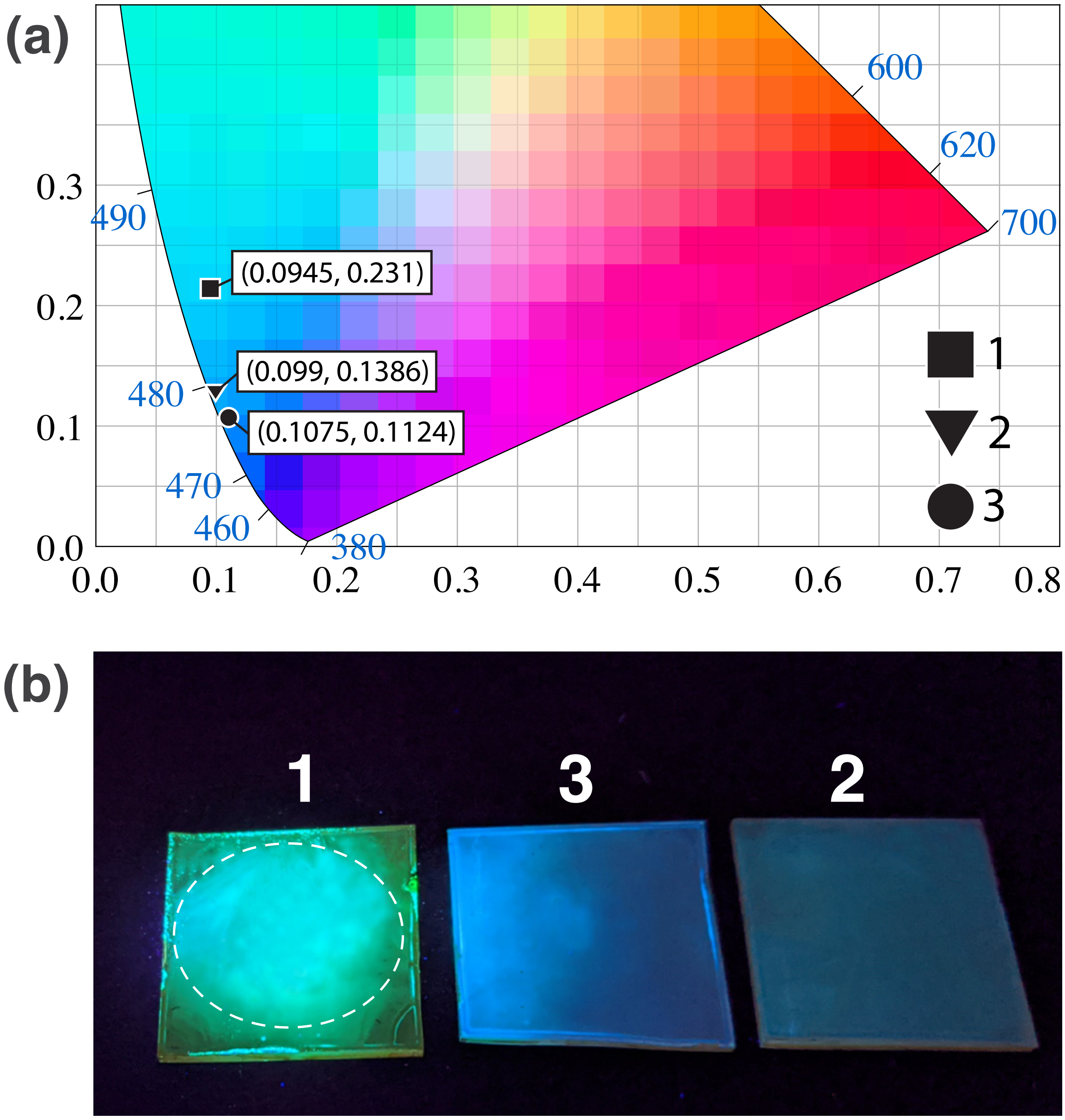

Figure S 2. (a) CIE 1931 coordinates displaying a color of photoluminescence from **1-3**. (b) A photograph of the sample **1** under intense UV light of 1 W·cm^{-2}.

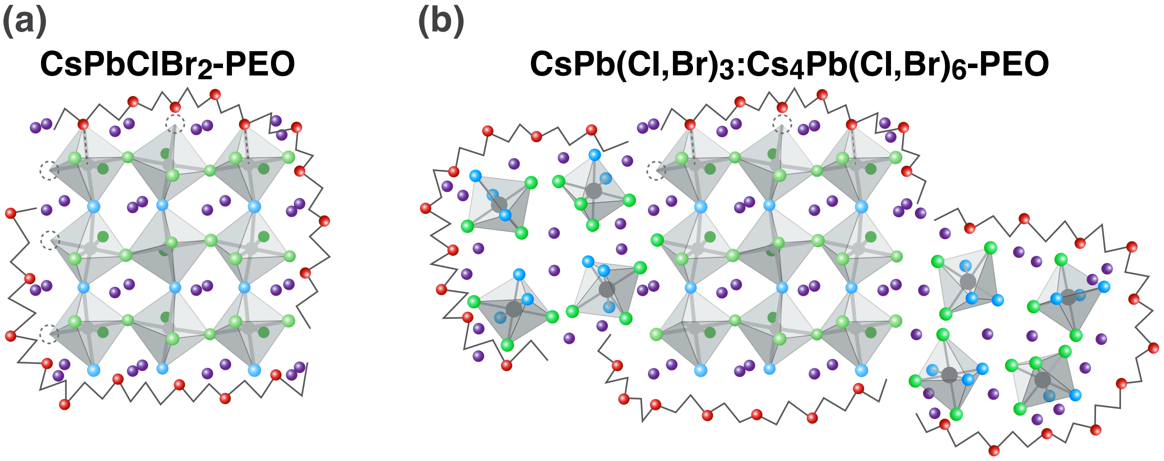

Figure S 3. (a) Partial passivation of surface halide vacancies in a perovskite grain by oxygen atoms of PEO. (b) More complete passivation of surface halide vacancies in a perovskite grain by both PEO and hexahalide crystallites.

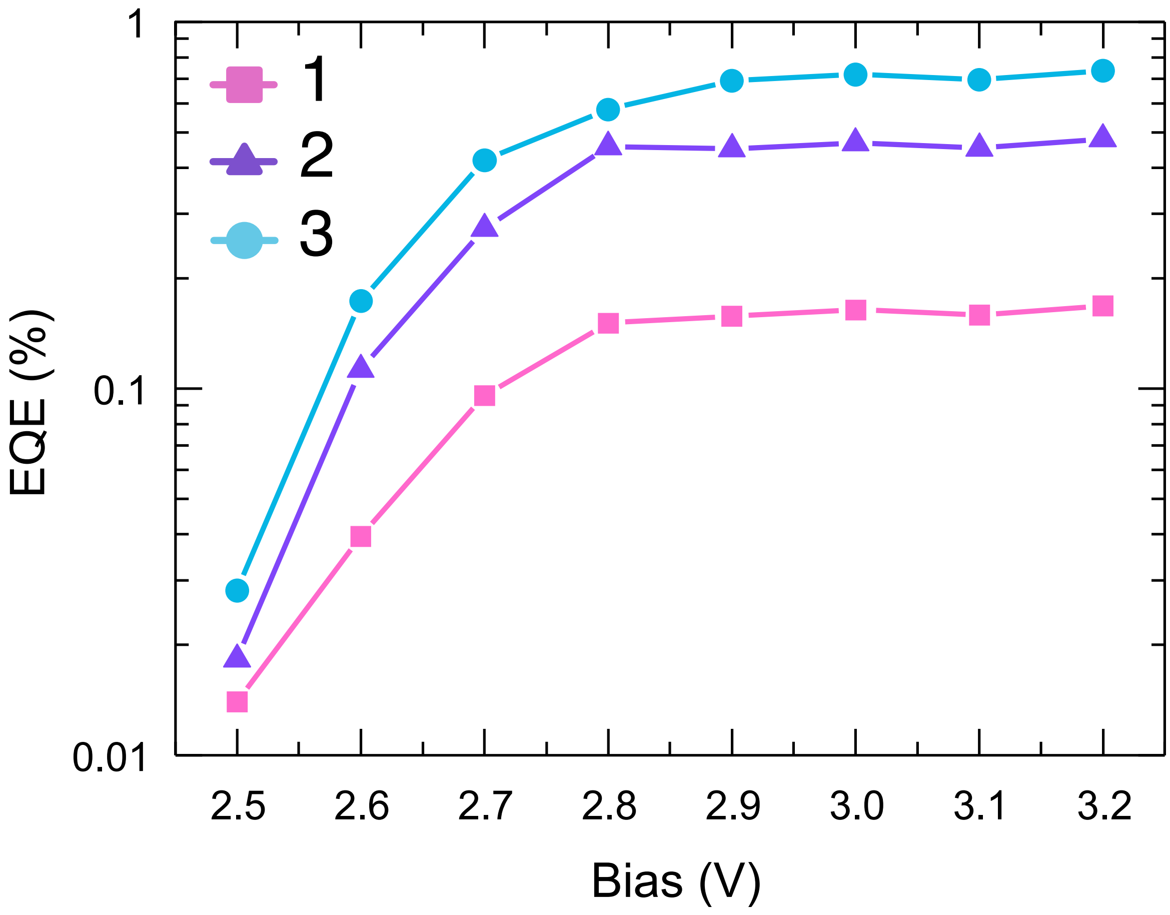

Figure S 4. Electroluminescence external quantum efficiency vs bias relations for the PeLECs **1**-**3**.
